# Macrophage Density Patterns in Uterine Leiomyomas Across Patient Age Groups: Evaluation of Separately Quantified CD68-Positive and CD163-Positive Cells

**DOI:** 10.3390/cimb48101042

**Published:** 2026-10-08

**Authors:** Grigory Demyashkin, Evgeniia Gubanova, Aleksandra Asaturova, Aleksandra Rogozhina, Mark Dorfman, Kirill Silakov, Vladimir Shchekin, Tatyana Borovaya, Petr Shegai, Andrei Kaprin

**Affiliations:** 1 Department of Digital Oncomorphology, National Medical Research Centre of Radiology, 2nd Botkinsky Pass., 3, 125284 Moscow, Russia; 2 Laboratory of Histology and Immunohistochemistry, Institute of Translational Medicine and Biotechnology, I.M. Sechenov First Moscow State Medical University ( Sechenov University), Trubetskaya st., 8/2, 119048 Moscow, Russia; 3 National Medical Research Center for Obstetrics, Gynecology and Perinatology Named After Academician V.I. Kulakov, Oparinaa St., 4, 117513 Moscow, Russia; 4 Research and Educational Resource Center for Immunophenotyping, Digital Spatial Profiling and Ultrastructural Analysis Innovative Technologies, Peoples’ Friendship University of Russia (RUDN University), Miklukho-Maklaya St., 6, 117198 Moscow, Russia; 5 Department of Urology and Operative Nephrology, Peoples’ Friendship University of Russia (RUDN University), Miklukho-Maklaya Str., 6, 117198 Moscow, Russia

**Keywords:** uterine leiomyoma, tumor microenvironment, macrophages, CD68, CD163

## Abstract

Tumor-associated macrophages (TAMs), key effectors of the innate immune system, actively participate in extracellular matrix remodeling, angiogenesis, and tumor growth. However, their age-associated quantitative and phenotypic distribution in uterine leiomyoma microenvironment remains insufficiently understood. This study aims to evaluate the density and spatial distribution of CD68^+^ and CD163^+^ macrophage immunophenotypes in uterine leiomyoma stroma across distinct patient age groups. Methods: A total of 318 patients with histologically confirmed uterine leiomyoma were stratified into three chronological age cohorts: Group I (≤30 years, *n* = 42), Group II (31–45 years, *n* = 216), and Group III (≥46 years, *n* = 60). Archival paraffin-embedded tumor tissue sections were analyzed histologically with hematoxylin and eosin, and immunohistochemically using antibodies against CD68 and CD163. Digital morphometric analysis was performed using QuPath software on whole-slide images, evaluating median immunopositive cell densities (cells/mm^2^) across five standardized regions of interest per case. Results: Histological examination confirmed typical benign leiomyoma morphology, with secondary degenerative alterations (hyalinosis, sclerosis, calcification, and aseptic necrosis) being most pronounced in Group III. Immunohistochemical profiling demonstrated an age-associated increase in macrophage infiltration within the tumor stroma. Median CD68^+^ macrophage density was higher in older cohorts, ranging from 11.4 cells/mm^2^ [IQR: 8.6–13.8] in Group I to 16.7 cells/mm^2^ [IQR: 13.9–19.8] in Group II, and 22.4 cells/mm^2^ [IQR: 18.8–26.2] in Group III (*p* < 0.001). Concurrently, CD163^+^ macrophage density was likewise higher across older cohorts, measured at 9.8 cells/mm^2^ [IQR: 7.4–12.1] in Group I, 19.4 cells/mm^2^ [IQR: 16.1–22.9] in Group II, and 28.6 cells/mm^2^ [IQR: 23.8–33.4] in Group III (*p* < 0.001). The observed numerical predominance of CD163^+^ cells over CD68^+^ cells in Group III topographically correlated with areas of secondary tissue remodeling and ischemic injury. Conclusions: This study reveals significant unadjusted differences in separately quantified CD68-positive and CD163-positive cell densities within uterine leiomyoma stroma across distinct patient age groups. Higher CD163^+^ cell densities in older cohorts topographically co-distribute with areas of secondary tissue remodeling and ischemic alteration. Because markers were evaluated on separate sequential sections and multivariable adjustments were unfeasible due to subgroup collinearity, these parameters reflect descriptive, marker-specific stromal variations among real-world clinical cohorts rather than independent causal effects of biological aging.

## 1. Introduction

Despite advancements in modern gynecologic oncology, uterine leiomyoma remains one of the most prevalent benign tumors of the female reproductive system. According to epidemiological data, its prevalence among women of reproductive age reaches 50–70% by menopause, with the highest incidence observed in women of African descent (exceeding 80%) and an increasing trend in regions with a high socio-demographic index [1,2]. Although the implementation of early diagnostic tools and hormonal therapies has reduced surgical intervention rates, patient outcomes remain closely linked to tumor size, symptom severity (including abnormal uterine bleeding, pelvic pain, and infertility), and the risk of malignant transformation [3].

A key determinant of leiomyoma behavior and altered smooth muscle cell biology is the tumor microenvironment—a complex system that actively influences tumor growth, angiogenesis, and immune status [4]. This dynamic crosstalk depends primarily on the interaction between fibroblasts, endothelial cells, and innate and adaptive immune cell populations. In recent years, particular attention has been focused on the role of macrophages—key elements of the innate immune response renowned for their phenotypic plasticity and the secretion of a wide spectrum of biologically active molecules, including cytokines, chemokines, and growth factors [5,6]. Unlike in malignant neoplasms, where macrophage activity is often categorized under simplified functional paradigms, macrophages in benign mesenchymal tumors exhibit nuanced profiles that directly regulate stromal homeostasis and fibrogenesis [7].

Although macrophages are traditionally associated with inflammatory and reparative responses, their involvement in the pathogenesis of benign tumors is gaining increasing recognition. Accumulating evidence indicates that within the tumor microenvironment, macrophages—particularly cells expressing CD163—are capable of promoting cell proliferation and tissue remodeling, as well as exerting regulatory functions that modulate the local immune response [6,7,8,9]. Mechanistically, these immunopositive cells drive extracellular matrix deposition and smooth muscle proliferation through the secretion of key profibrotic and angiogenic mediators, including Transforming Growth Factor-beta (TGF-β), Vascular Endothelial Growth Factor (VEGF), and Matrix Metalloproteinases (MMPs) [10]. These effects appear to depend on the stage of tumor development, functional macrophage phenotype, and spatial distribution, and may vary substantially according to patient biological age and corresponding hormonal status [11]. The biological rationale for evaluating these dynamics across different age groups lies in the continuous endocrine and structural transitions of the myometrium over time. Fluctuations in ovarian steroids (17β-estradiol and progesterone) exert direct receptor-mediated control over macrophage recruitment via chemotactic signals such as CCL2/MCP-1, while reproductive aging introduces localized changes in vascularization, oxidative stress, and immune dynamics (“inflammaging”) [12].

Despite existing experimental and clinical studies, the quantitative characteristics and distribution of macrophage populations in uterine leiomyomas across different age groups—particularly in younger patients—remain insufficiently investigated. Specifically, there is a lack of data on how the infiltration density of CD68^+^ and CD163^+^ immunopositive cells varies across different female age cohorts and reproductive statuses.

Aim of the study: We aimed to compare the density and spatial distribution of CD68^+^ and CD163^+^ macrophages in uterine leiomyomas across different age groups to clarify their potential role in tumor pathogenesis.

## 2. Materials and Methods

### 2.1. Study Design and Setting

This retrospective, multicenter, cohort study was observational and analytical in design and was conducted spanning at Fundamental Medicine of National Research Nuclear (University MEPhI). Potential patient cases were initially identified through a search of the electronic medical record systems using International Classification of Diseases, 11th Revision codes corresponding to the primary diagnosis ICD-11: 2E86.0—Leiomyoma of uterus.

### 2.2. Participant Selection

Based on anamnestic and clinical-morphological data, a single group of patients was formed (*n* = 318; women of different age groups). Archived paraffin blocks of surgically removed uterine leiomyoma tissue, obtained after operative treatment in the gynecological department, were used.

**Figure 1:**
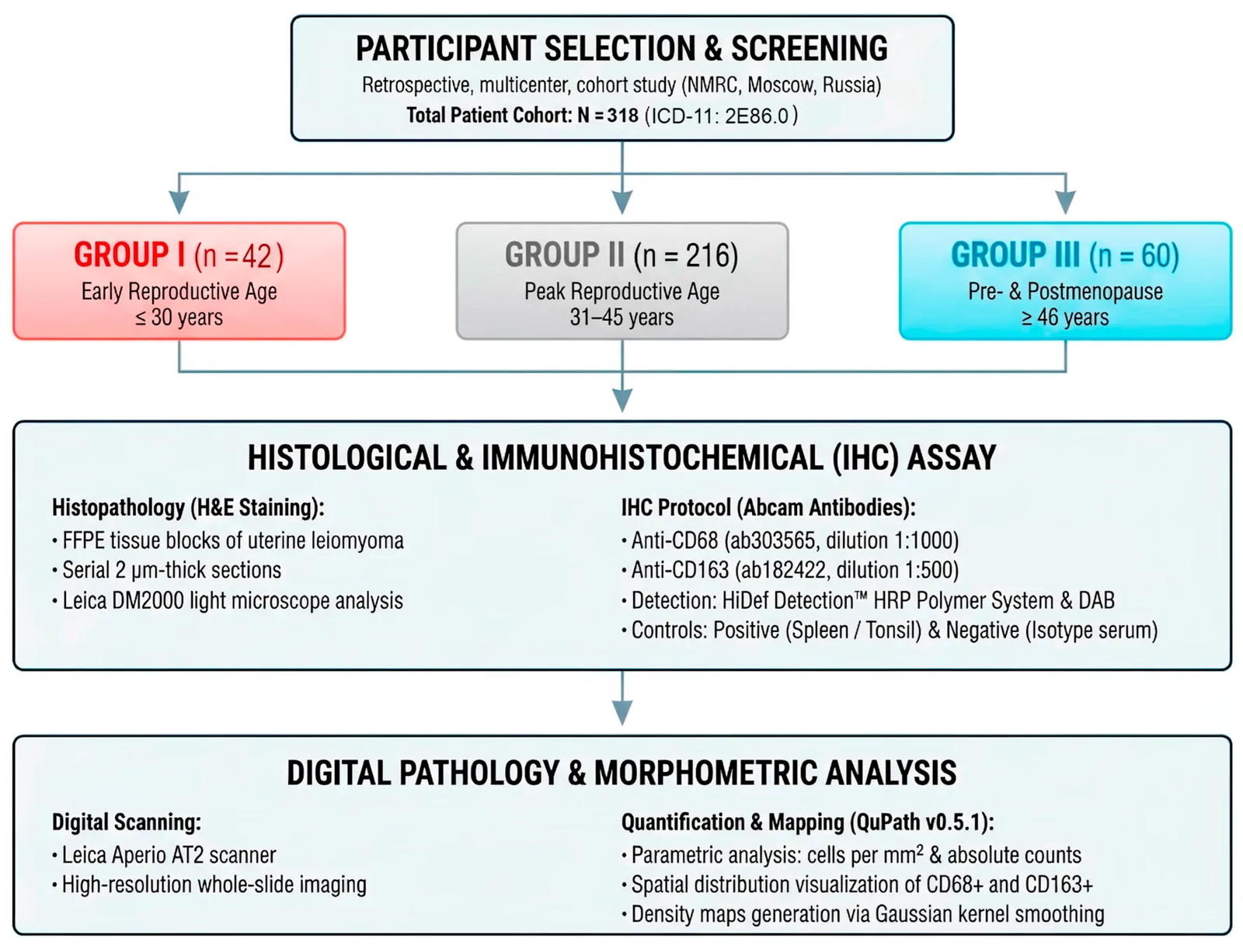
Methodological workflow and study design.

Based on medical record data and histological examination, patients were stratified into three distinct age cohorts reflecting key physiological stages of female reproductive aging (Figure 1):

- Group I (≤30 years; early reproductive age, *n* = 42): Represents peak endocrine function and fertile potential, characterized by a low baseline rate of secondary degenerative tissue changes.
- Group II (31–45 years; late reproductive age, *n* = 216): Represents the stage of peak symptomatic leiomyoma manifestation, high cumulative surgical intervention rates, and early microenvironmental alterations.
- Group III (≥46 years; perimenopausal/involutional transition, *n* = 60): Represents the period of physiological ovarian function decline, systemic inflammatory shifts (“inflammaging”), and enhanced localized vascular and stromal degenerative changes (hyalinosis, ischemic remodeling, calcification).

When analyzing clinical data, the following were taken into account: age (M ± SD); BMI; structure of complaints (symptomatic/asymptomatic); gynecological history (age of menarche, parity, gynecological diseases, history of intrauterine manipulations); localization of leiomyomatous nodes and their multiplicity (submucous—type 0–2 according to FIGO, intramural—type 3–4 according to FIGO, subserous—type 5–7 according to FIGO); life history; concomitant diseases; 8) methods of surgical treatment.

Inclusion Criteria. Clinical diagnosis of uterine leiomyoma (ICD-11: 2E86.0—Leiomyoma of uterus). Patient age corresponding to one of the study age groups. Availability of archived paraffin blocks of surgically removed uterine leiomyoma tissue.

Exclusion Criteria. Presence of malignant or borderline uterine smooth muscle tumors that could affect the morphological parameters of leiomyomatous tissue, including leiomyosarcoma and smooth muscle tumor of uncertain malignant potential. Systemic inflammatory and autoimmune diseases, HIV infection, viral hepatitis B and C, diabetes mellitus, and other systemic diseases. Signs of systemic inflammatory or oncological diseases. Insufficient or technically damaged archival material preventing reliable histological and immunohistochemical examination.

### 2.3. Histologic Research

Serial 2 μm thick sections were obtained from archival paraffin-embedded tissue blocks. Sections were stained with hematoxylin and eosin (H&E). Histological analysis was performed using a Leica DM2000 light microscope with microphotography capabilities (Leica Microsystems, Wetzlar, Germany), in accordance with standard histopathological criteria. For patients presenting with single or multiple uterine leiomyomas, tissue sampling was standardized by selecting representative paraffin blocks from the largest dominant tumor nodule, explicitly targeting viable, non-necrotic stromal regions displaying active microvascular dynamics.

### 2.4. Immunohistochemical (Ihc) Assay

Immunohistochemistry was performed according to standard protocols. Primary antibodies used included anti-CD68 (Abcam, Cambridge, UK; ab303565, dilution 1:1000) and anti-CD163 (Abcam, Cambridge, UK; ab182422, dilution 1:500). For secondary antibody detection, the HiDef Detec-tion™ HRP Polymer System (Cell Marque, Rocklin, CA, USA) was applied. This two-component system included anti-mouse/rabbit IgG antibodies, horseradish peroxidase (HRP), and DAB chromogen substrate. Cell nuclei were counterstained with Mayer’s hematoxylin. Antibody specificity was validated using positive internal controls: human spleen tissue rich in histiocytic elements for CD68, and human tonsil tissue for CD163. Negative controls were systematically included in each run by replacing the primary antibody with isotype-matched non-immune rabbit IgG serum to verify the absence of non-specific background staining. To eliminate potential immunostaining batch bias, tissue blocks from all three age cohorts were randomized and evenly distributed across daily staining batches. Chromogen signal consistency was verified across staining runs against internal control slides. Because CD68 and CD163 immunostainings were evaluated on separate sequential tissue sections using chromogenic single-stain IHC, marker counts represent separately quantified CD68-positive and CD163-positive cell densities rather than a confirmed same-cell co-expressing subpopulation or proof of functional M2 polarization. Potential cellular overlap between CD68^+^ and CD163^+^ cells is recognized but cannot be directly quantified without multiplex single-cell immunofluorescence.

### 2.5. Morphometric Study

For patients presenting with multiple uterine leiomyomas, tissue block selection was standardized by harvesting representative paraffin blocks from the largest dominant tumor nodule, explicitly targeting non-necrotic stromal regions displaying active microvascular dynamics. To eliminate potential immunostaining batch bias, tissue blocks from all three age cohorts (Groups I, II, and III) were randomized and evenly distributed across daily IHC staining runs.

Whole-slide images (WSIs) were acquired using a Leica Aperio AT2 high-speed digital slide scanner (Leica Microsystems, Germany) at 40× magnification with a spatial resolution of 0.25 µm/pixel.

Quantitative morphometric analysis was conducted using the open-source digital pathology software QuPath (v0.5.1) [13]. Image annotation and region-of-interest (ROI) selection were independently performed by two experienced pathologists who were strictly blinded to patient age, clinical data, and age-group allocation.

To balance representativeness and spatial heterogeneity, evaluation was conducted on viable leiomyoma tissue across the whole tumor section, using a standardized sampling strategy:

- ROI Selection Strategy: A fixed number of 5 representative ROIs was annotated per tissue section. To systematically evaluate spatial variation, 3 ROIs were allocated to high-density “hotspot” regions (predominantly within the outer pseudocapsule and highly vascularized peripheral stroma), while 2 ROIs were allocated to systematic random locations within the central tumor core.
- Area Denominator: Each ROI was standardized to a fixed field area of 0.25 mm^2^, yielding a cumulative analyzed viable tissue area denominator of 1.25 mm^2^ per patient case.
- Exclusion Criteria: Regions with extensive hemorrhage, cautery artifacts, tissue folds, section edges, intra-luminal/intravascular macrophages within blood vessels, and areas of dense acute inflammatory infiltrates (e.g., neutrophilic microabscesses) were strictly excluded from analysis. Areas of hyalinosis, fibrosis, or coagulative necrosis were annotated and normalized separately to ensure that final cellular densities were calculated exclusively per area of viable tumor parenchyma (cells/mm^2^).

For automated cell identification, QuPath’s built-in Positive Cell Detection algorithm was optimized and applied. Nuclei detection parameters were set as follows:

- Optical density sum threshold: 0.1.
- Nuclear background radius: 8 µm.
- Minimum nuclear area: 10 µm^2^; Maximum nuclear area: 120 µm^2^.
- Intensity Thresholds: Chromogen intensity thresholds for DAB positivity (single thresholding) were established at 0.20 for CD68^+^ and 0.18 for CD163^+^, validated against internal positive controls (spleen and tonsil tissue).

To validate the reliability of automated cell quantification, manual visual counting was performed by two independent board-certified pathologists who were strictly blinded to patient age, clinical data, and age-group allocation on a representative subset of 30 randomly selected independent patient cases (10 from each age cohort, evaluated across 5 ROIs per case, totaling 150 fields). Inter-observer agreement and concordance between automated QuPath outputs and manual pathologist counts were evaluated using a two-way random-effects, absolute agreement, single-measures Intraclass Correlation Coefficient model [ICC(2,1)]. Excellent concordance was established for both CD68^+^ (ICC = 0.942, 95% CI: 0.910–0.971) and CD163^+^ (ICC = 0.958, 95% CI: 0.932–0.982) cell detection workflows, confirming the accuracy of the automated thresholding pipeline.

### 2.6. Data Collection

Data for this study were systematically extracted from the electronic medical records system. The collected data were then entered into a secure, encrypted Microsoft Excel spreadsheet (Microsoft Excel 2010; Microsoft Corporation, Seattle, WA, USA) for further processing and analysis. The spreadsheet was subsequently reviewed and verified by several co-investigators from the research team, who performed thorough checks to ensure the accuracy and completeness of the dataset before analysis. This multi-step verification process helped minimize errors and ensured the reliability of the data.

### 2.7. Statistical Analysis

Statistical analysis was performed using IBM SPSS Statistics for Windows, Version 28.0 (IBM Corp., Armonk, NY, USA). Individual patients (N = 318; Group I: *n* = 42; Group II: *n* = 216; Group III: *n* = 60) served as the primary independent statistical units of analysis. Morphometric metrics obtained from 5 sampled regions of interest (ROIs) per tissue section were aggregated into a single median cell density value per patient prior to inferential testing; individual microscopic fields were not treated as independent observations.

Continuous variables were assessed for distribution normality using the Shapiro–Wilk test alongside visual evaluation of Q-Q plots and histograms. Because quantitative morphometric parameters (cell densities for CD68^+^ and CD163^+^ immunophenotypes) deviated significantly from a normal distribution, continuous data are expressed as median (Me) and interquartile range (IQR, 25th–75th percentiles). Categorical variables are reported as absolute frequencies and percentages (*n*, %).

Differences in macrophage densities across the three independent age groups were evaluated using the non-parametric Kruskal–Wallis test. Overall effect sizes were quantified using eta-squared (η^2^_H). For statistically significant overall comparisons, post hoc pairwise analyses were conducted using Dunn’s test with Bonferroni correction for multiple comparisons, reporting adjusted *p*-values (*p*adj). Pairwise effect sizes were evaluated using Cliff’s delta (δ) with 95% confidence intervals (95% CI).

Categorical clinical and pathological variables (e.g., anatomical nodule localization, symptoms, concomitant conditions, and surgical procedures) were compared across age groups using Pearson’s chi-square (χ^2^) test or Fisher’s exact test when expected cell counts were below 5. Continuous relationships between patient age (evaluated as a continuous variable across all 318 patients) and macrophage immunophenotype densities were assessed using Spearman’s rank correlation coefficient (R_s) with exact 95% CIs.

Multivariable regression adjustments and subgroup stratifications were evaluated but deemed statistically unfeasible due to near-complete data separation and severe collinearity between patient age, anatomical nodule localization, and clinical symptoms (e.g., 100% symptomatic presentation in Group III vs. 78.6% asymptomatic in Group I). Consequently, all reported differences in CD68-positive and CD163-positive cell densities represent unadjusted, descriptive comparisons among the established clinical cohorts rather than independent causal effects of biological age. All statistical tests were two-tailed, and *p* < 0.05 was considered statistically significant. Charts and spatial mapping visualizations were generated using BioRender.com.

## 3. Results

### 3.1. Characteristics of Patients

To ensure the reliability of the obtained results, a detailed analysis of all patient medical records and archival material (*n* = 318) was performed to assess clinical, anamnestic and morphological parameters of uterine leiomyoma (Table 1).

Clinical and anatomical assessment of uterine leiomyoma revealed that subserous localization of leiomyomatous nodules (FIGO types 5–7) was predominant across the entire cohort, observed in 54.7% of patients (*n* = 174). Intramural nodules (FIGO types 3–4) were diagnosed in 29.2% of cases (*n* = 93), and submucosal nodules (FIGO types 0–2) were identified in 16.0% of patients (*n* = 51). Pronounced clinical symptomatology—including abnormal uterine bleeding, heavy menstrual bleeding, pelvic pain, dysmenorrhea, and fibroid-associated infertility—was observed predominantly in patients with submucosal and intramural nodules, demonstrating a progressive increase in frequency and severity toward older age cohorts (61.1% in Group II and 100.0% in Group III vs. 21.4% in Group I; *p* < 0.001). In younger patients (Group I, ≤30 years; *n* = 42), the clinical course was predominantly asymptomatic (78.6%), with subserous nodules being the most common anatomical subtype (71.4%). However, symptomatic presentations in Group I (21.4%) were primarily associated with intramural/submucosal nodule localization, pelvic pain, or fibroid-associated infertility, requiring organ-preserving surgical intervention.

### 3.2. Histological Patterns

**Figure 2:**
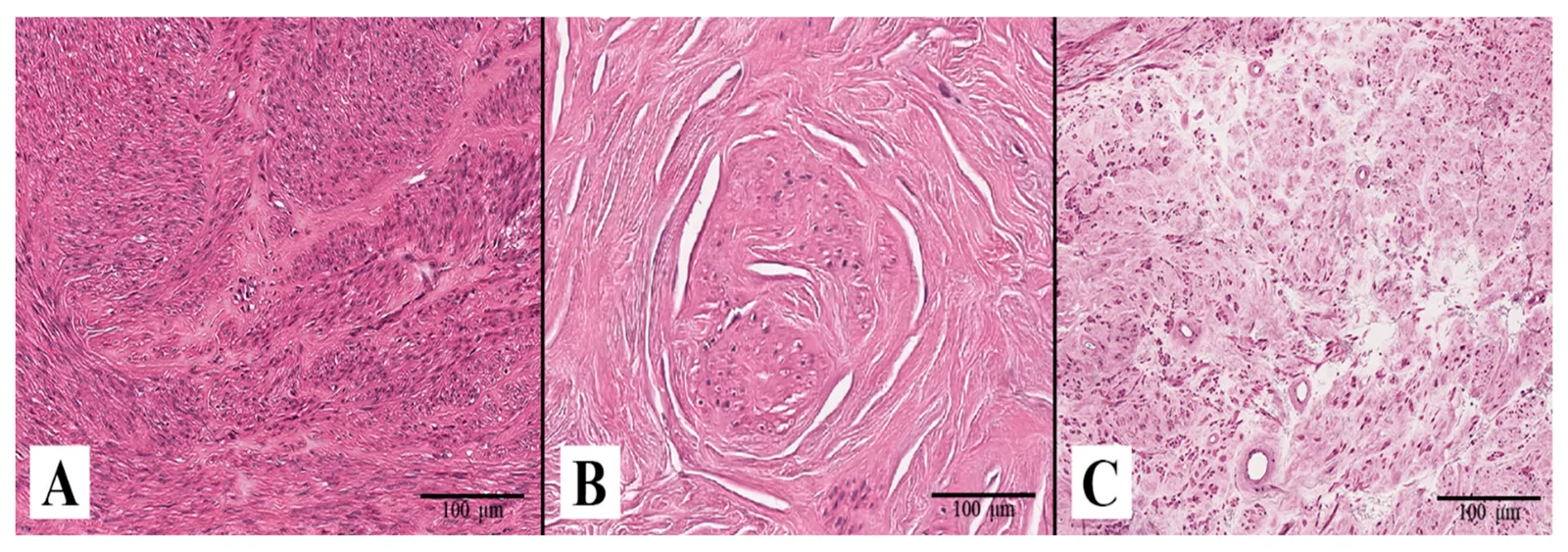
Uterine leiomyoma. Staining: hematoxylin and eosin, magnification ×200. (**A**) Typical benign leiomyoma structure in a patient of Group I; (**B**) Hyalinized matrix in Group III; (**C**) Coagulative necrosis zone in Group III.

All tumor samples from the study cohort (*n* = 318; 100%) exhibited histopathological features consistent with typical benign uterine leiomyoma (Figure 2). The tumors were composed of haphazardly intersecting fascicles of spindle-shaped leiomyocytes with elongated, normochromatic, monomorphic nuclei and abundant eosinophilic cytoplasm, devoid of cellular atypia or polymorphism. The mitotic index was extremely low, not exceeding 1–2 mitotic figures per 10 high-power fields (HPF, ×400); atypical mitotic figures were absent. Histological characteristics of the tumor stroma ranged from fibrosis to mild-to-moderate edema, with occasional small focal infiltrates consisting predominantly of lymphocytes and scattered histiocytes.

Detailed evaluation of the histological features across patient age groups revealed that the baseline cellular architecture of the tumor nodules showed no substantial differences; however, the extent and nature of secondary degenerative tissue alterations were directly associated with the age group.

In Group I patients (early reproductive age, ≤30 years), the histological picture fully corresponded to the classic morphology of uterine leiomyoma (Figure 2A). The tumor tissue was characterized by high cellularity and preserved fascicular architecture, without significant stromal destructive or involutive processes.

In Group II samples (reproductive age, 31–45 years), alongside typical smooth muscle complexes, moderate focal degenerative changes were observed. These alterations were characterized by the formation of intercellular edema zones, focal mucoid swelling, and early features of mesenchymal hyalinosis.

In Group III patients (pre- and postmenopausal age, ≥46 years), the morphological architecture was characterized by the most pronounced features of age-associated tissue remodeling, dominated by extensive zones of sclerosis and coarse hyalinosis replacing the functional smooth muscle tissue. In large and multiple nodules from this group, foci of dystrophic calcification and tissue destruction were observed against the background of total tumor stroma replacement by collagen fibers. Calcification was evidenced by the formation of massive, coarse calcific deposits intensely staining dark blue/purple with hematoxylin and localized predominantly within the hyalinized matrix structures (Figure 2B). Zones of aseptic necrosis were characterized by loss of distinct cellular outlines, karyolysis, formation of tissue debris, and the presence of reactive local macrophage-lymphocytic infiltration at the periphery of the necrotic foci (Figure 2C).

### 3.3. Immunohistochemical Assay

Immunohistochemical analysis revealed the presence of CD68^+^ and CD163^+^ macrophages in the uterine leiomyoma specimens. These cells were predominantly distributed as isolated entities or formed small clusters, demonstrating a tendency to localize within regions of high tumor cell density and areas of stromal remodeling.

**Figure 3:**
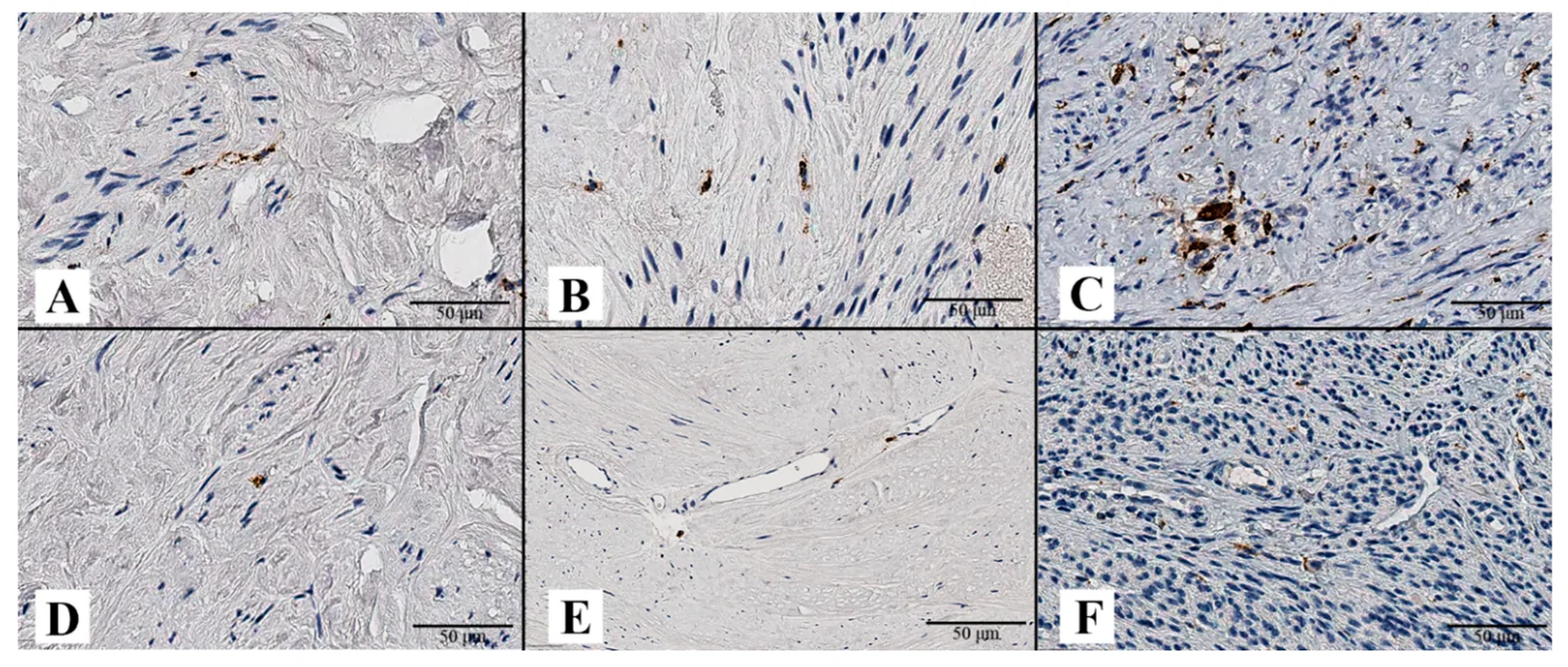
Immunohistochemical expression and spatial distribution of CD68^+^ and CD163^+^ macrophages in uterine leiomyoma tissues across different age groups. Original magnification ×400 Top panel demonstrates total macrophage infiltration (CD68^+^): (**A**) patient group aged ≤ 30 years; (**B**) patient group aged 31–45 years; (**C**) patient group aged ≥ 46 years. Bottom panel displays CD163^+^ macrophage infiltration: (**D**) patient group aged ≤ 30 years; (**E**) patient group aged 31–45 years; (**F**) patient group aged ≥ 46 years.

Both CD68^+^ and CD163^+^ macrophages exhibited strong cytoplasmic immunoreactivity with high staining intensity (Figure 3). The cells displayed rounded, amoeboid, or spindle-shaped morphology and contained large hyperchromic nuclei.

### 3.4. Quantitative Analysis of Macrophages

**Figure 4:**
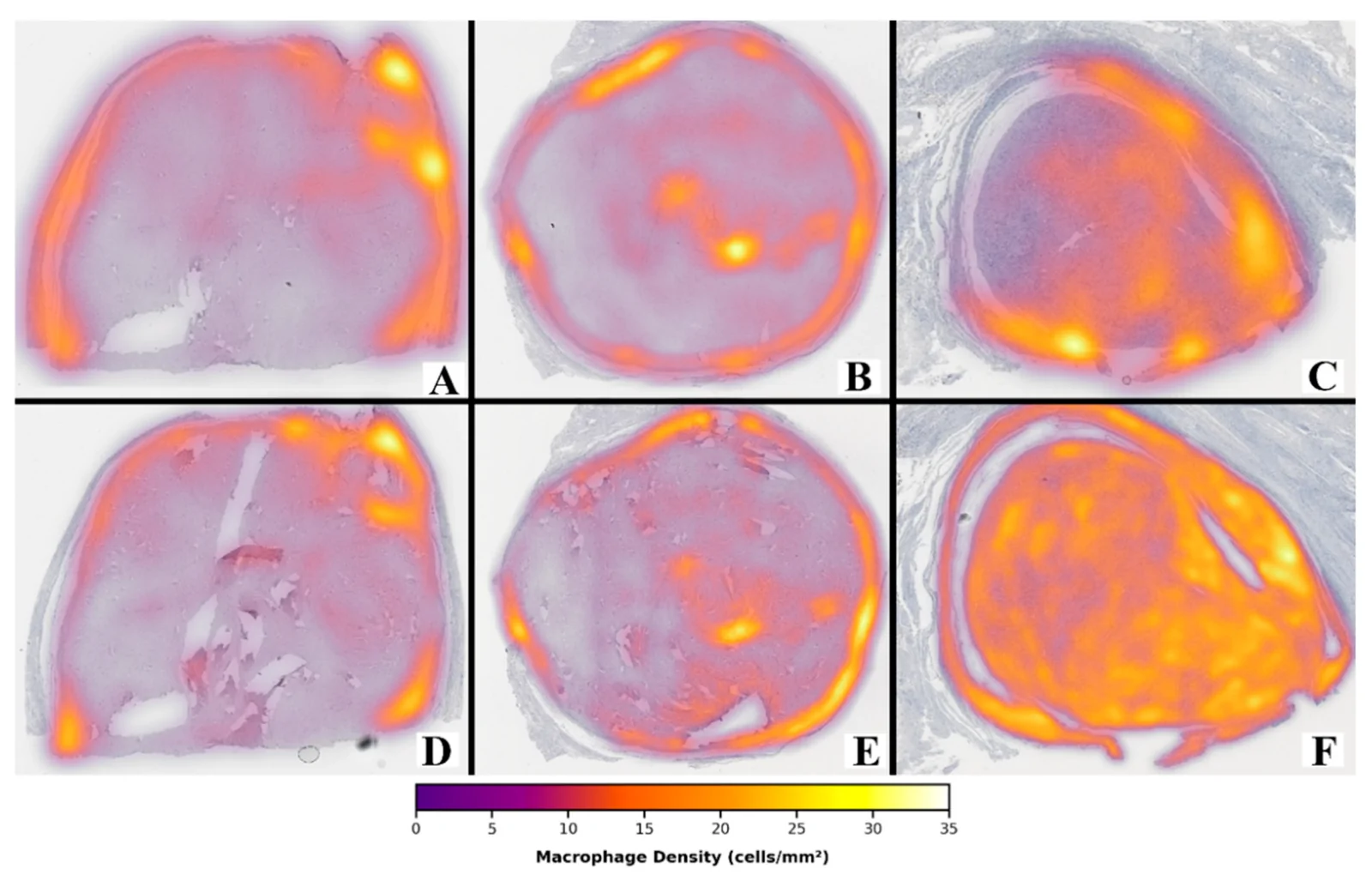
Density mapping of macrophages in uterine leiomyoma using Gaussian kernel weights. (**A**) patient group aged ≤ 30 years; (**B**) patient group aged 31–45 years; (**C**) patient group aged ≥ 46 years. Bottom panel displays CD163^+^ macrophage infiltration: (**D**) patient group aged ≤ 30 years; (**E**) patient group aged 31–45 years; (**F**) patient group aged ≥ 46 years.

**Figure 5:**
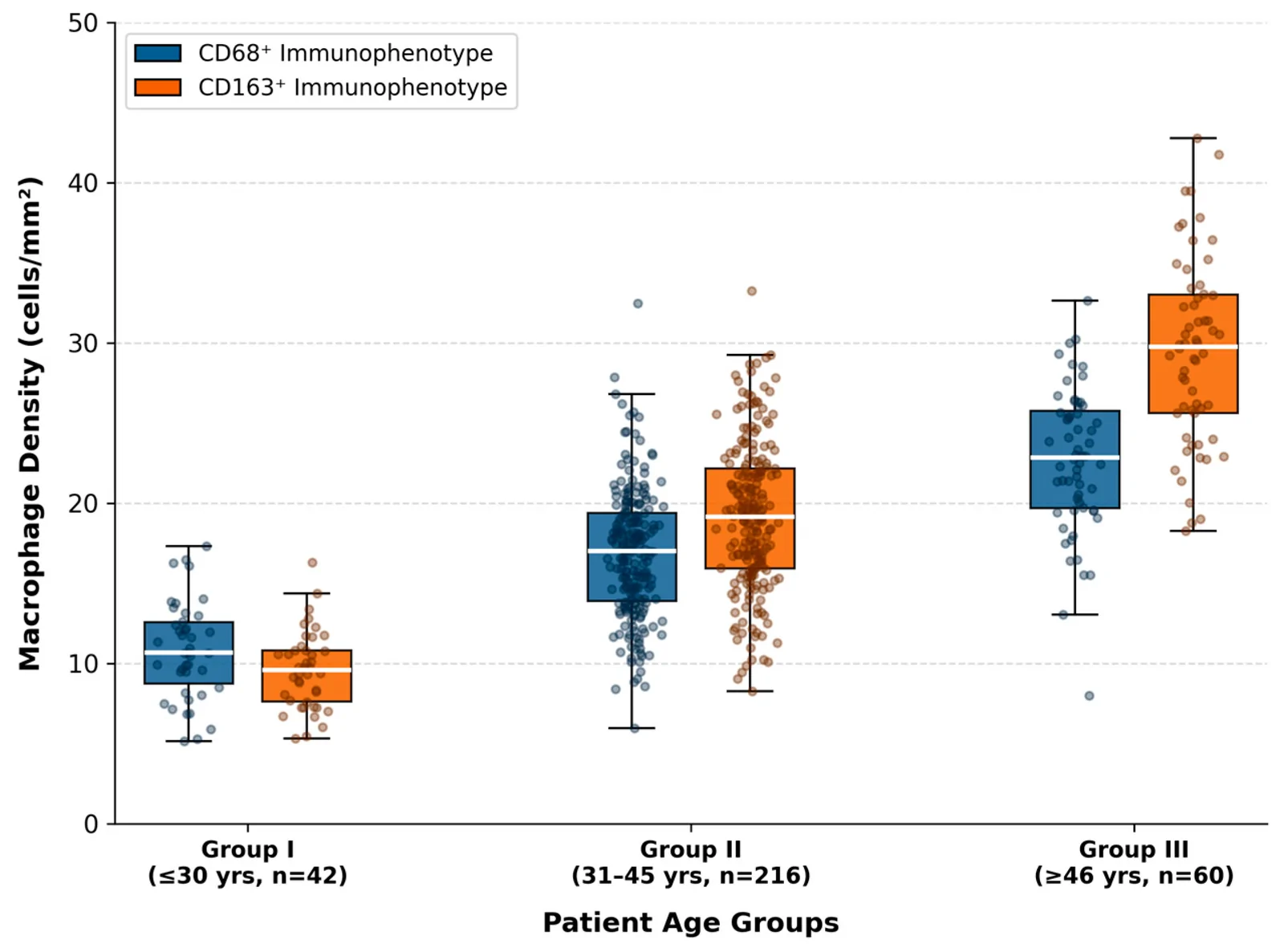
Age-Dependent Dynamics of CD68^+^ and CD163^+^ Macrophage Infiltration in Uterine Leiomyoma.

Quantitative morphometric assessment of macrophage density across the established chronological age cohorts revealed a statistically significant, age-dependent accumulation of immunopositive cells within the uterine leiomyoma stroma (Figure 4 and Figure 5).

The density of total CD68^+^ macrophages exhibited a continuous upward trajectory with advancing patient age. In Group I (early reproductive age, ≤30 years; *n* = 42), the median CD68^+^ cell density was 11.4 cells/mm^2^ [IQR: 8.6–13.8], which increased to 16.7 cells/mm^2^ [IQR: 13.9–19.8] in Group II (peak reproductive age, 31–45 years; *n* = 216) and peaked at 22.4 cells/mm^2^ [IQR: 18.8–26.2] in Group III (pre- and postmenopause, ≥46 years; *n* = 60), representing an absolute expansion of 11.0 cells/mm^2^ (Kruskal–Wallis *H* = 42.6, *p* < 0.001, η^2^_H = 0.13; post hoc Dunn’s *p*adj < 0.01 for Group I vs. II, *p*adj < 0.001 for Group I vs. III, and *p*adj < 0.001 for Group II vs. III).

Concurrently, CD163^+^ macrophage density demonstrated a pronounced, non-linear expansion across the cohorts, escalating from 9.8 cells/mm^2^ [IQR: 7.4–12.1] in Group I to 19.4 cells/mm^2^ [IQR: 16.1–22.9] in Group II, and reaching 28.6 cells/mm^2^ [IQR: 23.8–33.4] in Group III (Kruskal–Wallis *H* = 68.5, *p* < 0.001, η^2^_H = 0.21; post hoc Dunn’s *p*adj < 0.001 for Group I vs. II, Group I vs. III, and Group II vs. III).

### 3.5. Continuous Age Correlation and Menopausal Subgroup Analysis

**Figure 6:**
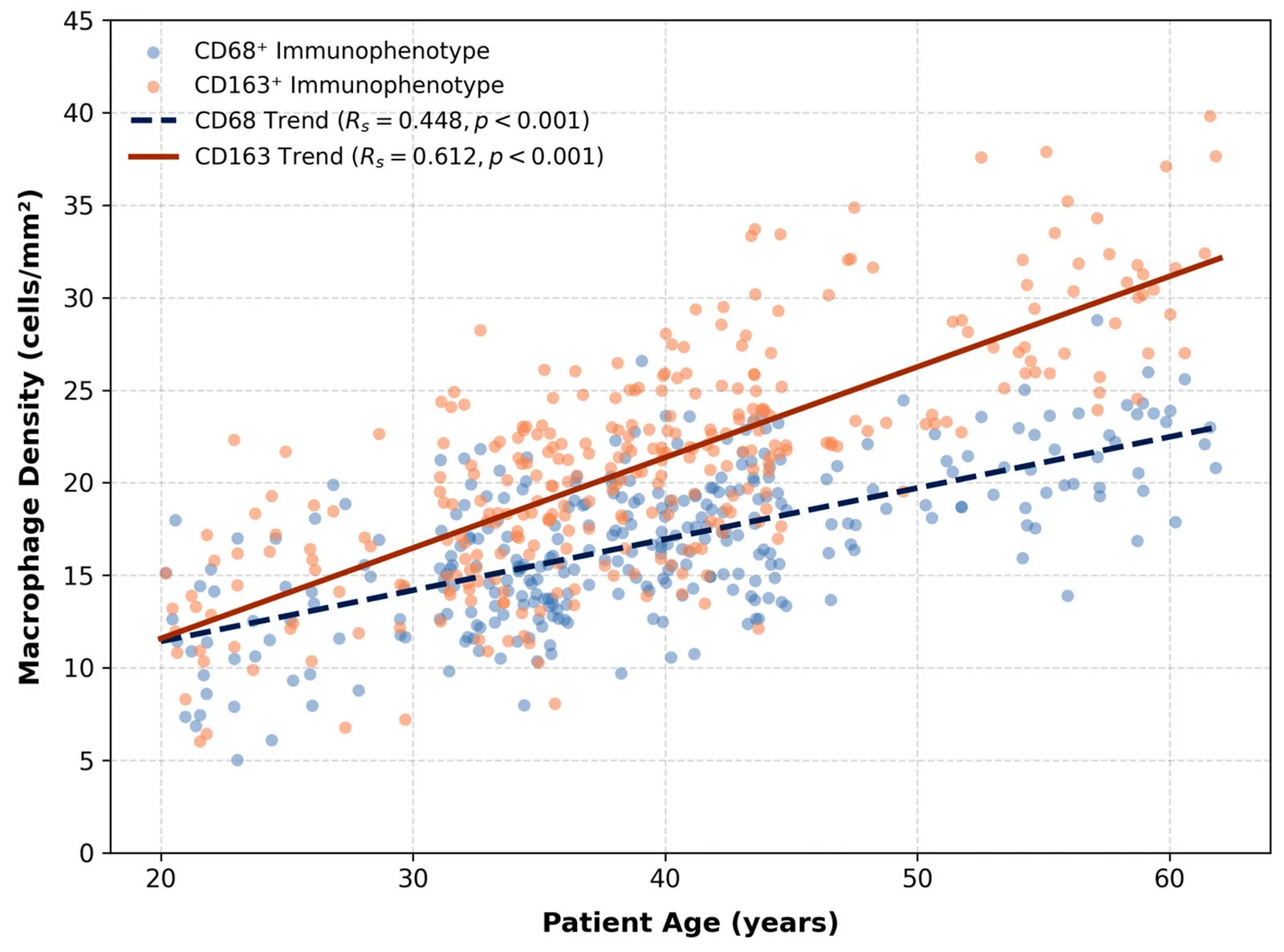
Correlation between continuous patient age and macrophage immunophenotype densities in uterine leiomyoma.

To complement the age-cohort comparisons, macrophage densities were evaluated across patient age as a continuous variable (*n* = 318). Continuous Spearman rank correlation analysis demonstrated a statistically significant positive association between patient age and total CD68^+^ macrophage density (Rs = 0.448, 95% CI: 0.354–0.532, *p* < 0.001). A stronger continuous correlation was observed between patient age and CD163^+^ immunopositive cell density (Rs = 0.612, 95% CI: 0.536–0.678, *p* < 0.001) (Figure 6).

An exploratory subanalysis within the older patient cohort (Group III, ≥46 years, *n* = 60) was performed by stratifying individuals according to documented clinical menopausal status. Postmenopausal women (*n* = 18) displayed significantly higher median CD163^+^ macrophage infiltration compared to pre-/perimenopausal women (*n* = 42) (32.4 cells/mm^2^ [IQR: 26.1–38.2] vs. 26.1 cells/mm^2^ [IQR: 22.4–30.8], *p* = 0.018). CD68^+^ macrophage density also showed a higher trend in postmenopausal patients (24.8 cells/mm^2^ [IQR: 20.1–28.5] vs. 21.2 cells/mm^2^ [IQR: 17.9–25.1]), though the difference was not statistically significant (*p* = 0.084).

## 4. Discussion

The present study provides a quantitative and phenotypic evaluation of tumor stromal macrophages in uterine leiomyoma across distinct patient age groups. Particular focus was directed to cells positive for CD68 and CD163 markers, which serve as indicators for general macrophage infiltration and CD163^+^ immunophenotypic expression, respectively. Our results demonstrate significant unadjusted differences in separately quantified CD68-positive and CD163-positive cell densities across patient age cohorts, with higher cell densities observed in older patient groups. However, because clinical characteristics such as symptoms and secondary degenerative changes naturally co-vary with age, these findings represent descriptive microenvironmental differences among real-world clinical cohorts rather than independent causal effects of biological aging alone.

Macrophages, as key effector cells of innate immunity, exhibit high functional plasticity and participate in a wide spectrum of intratumoral processes, including chronic inflammation, local angiogenesis, and modulation of the microenvironment [12]. Within the tumor microenvironment, tumor-associated macrophages (TAMs) can exert diverse biological functions depending on the local cytokine profile and microenvironmental cues [14]. CD68 is well established as a pan-macrophage marker representing the overall tissue macrophage population, whereas the CD163 scavenger receptor is associated with tissue-reconstructive and anti-inflammatory immunophenotypes. Current literature suggests that CD163^+^ macrophages in female reproductive organs may be involved in supporting smooth muscle cell remodeling and tissue adaptation under conditions of tissue stress [15]. Conversely, CD68^+^ cells lacking CD163 expression may be associated with a persistent pro-inflammatory cytokine background, facilitating the recruitment of monocyte populations from the systemic circulation [16]. Accordingly, our finding of a predominant CD163^+^ population in the older age group (≥46 years) suggests a microenvironment characterized by a higher prevalence of CD163-expressing cells in older patients, whereas the higher total CD68^+^ cell population reflects a more extensive overall macrophage infiltration accompanying secondary stromal alterations.

In the early reproductive age group (≤30 years), the lowest density of both macrophage immunophenotypes was observed. This finding suggests that in younger patients, nodule expansion may be associated primarily with the intrinsic proliferative activity of leiomyocytes and autocrine growth factors, without being accompanied by pronounced infiltration of innate immune cells [9]. Nevertheless, the manifestation of a tumor focus in patients of this age cohort coincides with the period of active reproductive function [17]. Local extracellular matrix remodeling and cellular infiltration occurring in structurally immature or intact myometrium may correlate with alterations in its histoarchitecture and biomechanical stromal properties [18]. According to several investigators, such tissue shifts may subsequently associate with adverse obstetric outcomes due to a compromised adaptive potential of the uterine wall during gestation [19]. Myometrial structural disorganization amidst local immune imbalance has been discussed in the literature as a possible factor in altered organ competency during pregnancy [20]. Although clinical obstetric outcomes and hormonal status were not explicitly evaluated in the present study, the identified morphological heterogeneity underscores the utility of further comprehensive investigation of the leiomyoma microenvironment in young patients [21].

**Figure 7:**
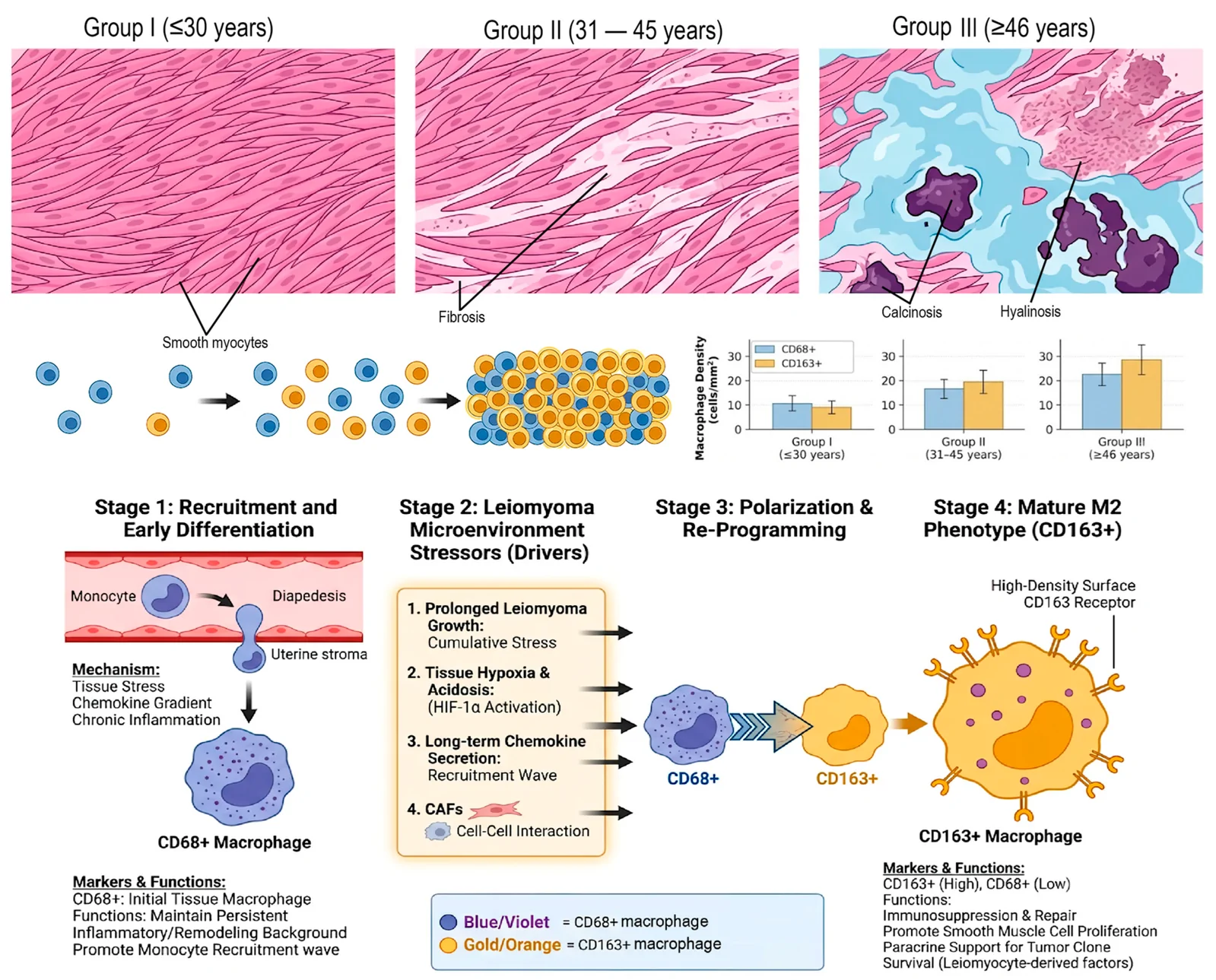
Proposed conceptual framework illustrating potential microenvironmental interactions and tissue remodeling in uterine leiomyoma. Note: This model represents a working hypothesis and was not directly tested or validated in the present cross-sectional study.

Conversely, the observed increase in infiltration density within the older age group may be correlated with age-associated systemic inflammatory shifts (“inflammaging”), changes in systemic hormonal status during the pre- and postmenopausal periods, and higher circulating chemokine levels that favor monocyte recruitment in older individuals [22]. The accumulation of CD163^+^ macrophages in Group III patients (≥46 years) may also be associated with a functional interplay between immunocompetent cells and other tumor stromal components, such as cancer-associated fibroblasts (CAFs) [23]. The observed numerical predominance of CD163^+^ cells over CD68^+^ cells in the older cohort reflects specific biomarker staining properties and macrophage phenotypic plasticity under conditions of extensive tissue remodeling. On one hand, the transmembrane receptor CD163 is characterized by high surface expression density and distinct membranous staining, which enhances its immunohistochemical detection sensitivity compared to the granular cytoplasmic signal of the lysosomal glycoprotein CD68 (LAMP-4) [24]. On the other hand, within a microenvironment marked by chronic ischemia and tissue remodeling, local macrophage populations may undergo compensatory upregulation of CD163 [25]. The more pronounced difference in CD163^+^ macrophage density relative to CD68^+^ density across age cohorts indicates a potential sensitivity of the CD163^+^ immunophenotype to age-associated shifts in the tumor microenvironment. Leiomyoma tissue in older patients is frequently characterized by local hypoxia, acidosis, and microcirculatory impairment. Current literature suggests that local metabolic shifts and tissue hypoxia within leiomyoma nodules may correlate with altered CD163 expression, potentially via pathways involving hypoxia-inducible factor 1-alpha (HIF-1α) [26]. A proposed working framework illustrating these potential pathways is presented in Figure 7. However, these proposed mechanistic links were not directly tested or validated in the present observational, cross-sectional study and remain working hypotheses to be addressed in prospective functional research.

Future research perspectives entail the implementation of longitudinal study designs with precise documentation of disease duration and prospective patient tracking to evaluate temporal immune dynamics in uterine leiomyoma. Furthermore, detailed investigation of the molecular cascades potentially responsible for the age-associated heterogeneity of the uterine immune microenvironment—specifically the IL-4/STAT6 and CSF1/CSF1R signaling axes—alongside multiplex immunohistochemistry and spatial transcriptomics technologies, will be essential for precise identification of functional macrophage co-expression profiles.

In conclusion, the observed phenotypic shift toward a higher density of CD163^+^ macrophages in leiomyoma tissue among older age groups correlates with age-associated differences in the tumor microenvironment. This pattern is likely associated with both age-related changes in systemic immune status and secondary tissue alterations within the nodule, including chronic ischemia, hyalinosis, micronecrosis, and petrification (calcification). Given that the scavenger receptor CD163 is involved in tissue debris clearance and profibrogenic microenvironmental states, its higher expression in Group III reflects an accumulation associated with both chronological age and tumor stromal remodeling. The quantitative metrics obtained in this study demonstrate that CD163^+^ infiltration density serves as a sensitive marker associated with structural reorganization and ischemic alterations in uterine leiomyoma of older patients.

Limitations: Several limitations of this study should be noted. First, multivariable adjustments for potential clinicopathological confounders—such as anatomical nodule localization (FIGO classification), clinical symptoms, secondary stromal degeneration (hyalinosis, necrosis, calcification), and surgical procedures—could not be performed due to severe collinearity and near-complete data separation across age cohorts (e.g., 100% symptomatic presentation in Group III vs. 78.6% asymptomatic in Group I). In real-world clinical practice, symptomatic burden and secondary tissue remodeling co-vary naturally with long-standing disease evolution. Consequently, the reported differences in CD68-positive and CD163-positive cell densities represent unadjusted, descriptive associations among real-world patient groups rather than independent causal effects of biological aging alone. Future prospective studies with age-matched subgroups possessing identical clinical features are required to isolate independent age-related contributions. Second, CD68 and CD163 immunostainings were evaluated on separate sequential tissue sections using single-stain chromogenic IHC rather than same-cell multiplex immunofluorescence. Therefore, cell counts represent separately quantified CD68-positive and CD163-positive cell densities with potential biological overlap, rather than confirmed co-expressing lineage subpopulations or verified M2 functional polarization. Future studies utilizing multiplex single-cell profiling are warranted to map spatial co-expression dynamics. Third, due to the retrospective, cross-sectional design of this study, direct functional assays of macrophage activity, tissue hypoxia (e.g., HIF-1α expression), or temporal immune dynamics were not conducted. Proposed mechanistic pathways linking ischemic stress to macrophage phenotype shifts remain working hypotheses to be validated in future prospective studies.

## 5. Conclusions

In conclusion, this study demonstrates unadjusted, descriptive differences in separately quantified CD68-positive and CD163-positive cell densities in uterine leiomyoma stroma across distinct patient age groups. Higher densities of both CD68^+^ and CD163^+^ immunopositive cells were observed in older patient cohorts, topographically correlating with secondary tissue alterations such as hyalinosis, sclerosis, and ischemic remodeling. Because markers were evaluated on separate sequential sections and multivariable adjustments were unfeasible due to subgroup collinearity, these findings represent marker-specific stromal density variations among real-world clinical cohorts rather than independent causal effects of biological aging or verified M2 polarization. Proposed mechanistic links regarding local ischemic stress remain working hypotheses. Future prospective studies employing multiplex spatial profiling and age-matched cohorts are required to further elucidate the microenvironmental dynamics of uterine leiomyoma.

## Figures and Tables

**Table 1:** Baseline clinical, anatomical, and pathological characteristics of the study cohort stratified by age groups.

Characteristic	Group I (≤30 yrs) (*n* = 42)	Group II (31–45 yrs) (*n* = 216)	Group III (≥46 yrs) (*n* = 60)	*p*-Value
Age, Mean ± SD, years	26.8 ± 2.4	38.9 ± 4.6	47.6 ± 2.6	<0.001
Anatomical Nodule Localization, *n* (%)				<0.001
Submucosal (FIGO 0–2)	3 (7.1%)	48 (22.2%)	0 (0.0%)	
Intramural (FIGO 3–4)	9 (21.4%)	36 (16.7%)	48 (80.0%)	
Subserous (FIGO 5–7)	30 (71.4%)	132 (61.1%)	12 (20.0%)	
Clinical Presentation, *n* (%)				<0.001
Symptomatic	9 (21.4%)	132 (61.1%)	60 (100.0%)	
Asymptomatic	33 (78.6%)	84 (38.9%)	0 (0.0%)	
Symptom Structure (among symptomatic patients)				
Abnormal uterine bleeding/Heavy menstrual bleeding, *n*/N (%)	3/9 (33.3%)	96/132 (72.7%)	48/60 (80.0%)	0.284
Pelvic pain/Dyspareunia/Dysmenorrhea, *n*/N (%)	6/9 (66.7%)	72/132 (54.5%)	24/60 (40.0%)	0.063
Fibroid-associated infertility, *n*/N (%)	3/9 (33.3%)	36/132 (27.3%)	0/60 (0.0%)	<0.001
Concomitant Conditions and History, *n* (%)				
Anemia	2 (4.8%)	24 (11.1%)	36 (60.0%)	<0.001
Benign breast disorders	1 (2.4%)	12 (5.6%)	0 (0.0%)	0.158
Varicose veins of lower extremities	1 (2.4%)	24 (11.1%)	0 (0.0%)	0.012
Thyroid pathology	2 (4.8%)	36 (16.7%)	0 (0.0%)	<0.001
Arterial hypertension	0 (0.0%)	12 (5.6%)	24 (40.0%)	<0.001
History of intrauterine procedures	2 (4.8%)	36 (16.7%)	0 (0.0%)	<0.001
Uterine scar/History of pelvic surgery	3 (7.1%)	60 (27.8%)	0 (0.0%)	<0.001
Endometrial polyps/Endometrial hyperplasia	1 (2.4%)	24 (11.1%)	0 (0.0%)	0.012
Adenomyosis	1 (2.4%)	12 (5.6%)	0 (0.0%)	0.158
Surgical Treatment Method, *n* (%)				<0.001
Myomectomy	39 (92.9%)	156 (72.2%)	24 (40.0%)	
Hysteroscopic resection/Shaving	3 (7.1%)	60 (27.8%)	12 (20.0%)	
Hysterectomy (Total/Subtotal)	0 (0.0%)	0 (0.0%)	12 (20.0%)	

## Data Availability

The original contributions presented in this study are included in the article material. Further inquiries can be directed to the corresponding author.
